# Converging Pathways: A Review of Pulmonary Hypertension in Interstitial Lung Disease

**DOI:** 10.3390/life14091203

**Published:** 2024-09-23

**Authors:** Alexandra Lawrence, Katherine Jane Myall, Bhashkar Mukherjee, Philip Marino

**Affiliations:** 1 Guy’s and St Thomas’ NHS Foundation Trust, London SE1 9RT, UK; alexandra.lawrence@gstt.nhs.uk (A.L.); katherinejane.myall@nhs.net (K.J.M.); bhashkar.mukherjee@gstt.nhs.uk (B.M.); 2 King’s College Hospital, London SE5 9RS, UK; 3 National Pulmonary Hypertension Service, Royal Brompton Hospital, London SW3 6NP, UK

**Keywords:** pulmonary hypertension, interstitial lung disease

## Abstract

Pulmonary hypertension (PH) in interstitial lung disease (ILD) is relatively common, affecting up to 50% of patients with idiopathic pulmonary fibrosis (IPF). It occurs more frequently in advanced fibrotic ILD, although it may also complicate milder disease and carries significant clinical implications in terms of morbidity and mortality. Key pathological processes driving ILD-PH include hypoxic pulmonary vasoconstriction and pulmonary vascular remodelling. While current understanding of the complex cell signalling pathways and molecular mechanisms underlying ILD-PH remains incomplete, there is evidence for an interplay between the disease pathogenesis of fibrotic ILD and PH, with interest in the role of the pulmonary endothelium in driving pulmonary fibrogenesis more recently. This review examines key clinical trials in ILD-PH therapeutics, including recent research showing promise for the treatment of both ILD-PH and the underlying pulmonary fibrotic process, further supporting the hypothesis of interrelated pathogenesis. Other important management considerations are discussed, including the value of accurate phenotyping in ILD-PH and the success of the “pulmonary vascular” phenotype. This article highlights the close and interconnected nature of fibrotic ILD and PH disease pathogenesis, a perspective likely to improve our understanding and therapeutic approach to this complex condition in the future.

## 1. Introduction

Interstitial lung diseases (ILD) describe a diverse range of pulmonary disorders, each characterised by varying degrees of interstitial inflammation, fibrosis, or a combination of both, resulting in lung tissue damage [1]. The aetiology of ILD is complex and thought to be multifactorial, with risk factors such as environmental or drug exposures, comorbid medical conditions such as connective tissue disease, and a familial or genetic predisposition frequently identified [2,3]. When no definitive cause can be determined the condition is classified as an idiopathic ILD [2]. The disease course and prognosis of ILD varies significantly between ILD subtypes, individuals, and their response to treatment. Those who develop progressive pulmonary fibrosis experience declining respiratory function, eventually leading to respiratory failure [4]. When ILD is further complicated by pulmonary hypertension (PH)—a condition characterised by elevated pressure and pathological changes within the pulmonary arteries—patients suffer from more severe symptoms, reduced exercise capacity, an increased need for supplemental oxygen, and a significant decline in both health-related quality of life and overall prognosis [5,6].

Although the mechanisms underlying ILD-PH are not fully understood, there are notable overlapping features with pulmonary arterial hypertension (PAH), including pulmonary artery endothelial dysfunction and vascular remodelling [7]. Despite these shared characteristics, the use of PAH-specific therapies has historically been ineffective at treating ILD-PH. The recent INCREASE study investigating an inhaled prostacyclin analogue showed modest benefit to both ILD-PH [8] and the underlying ILD in a post-hoc analysis [9], with further research currently underway [10]. This development has reignited interest in ILD-PH, leading experts to consider an overlapping disease pathogenesis and potentially reciprocal relationship between the closely related pulmonary vasculature and fibrotic pulmonary parenchyma [11,12].

This review will first place ILD-PH within the broader context of PH, then discuss the epidemiology, natural history, histopathology, and pathophysiology specific to ILD-PH. We examine the various cell signalling pathways and molecular mechanisms that suggest an interplay exists between the disease pathogenesis of fibrotic ILD and PH. This perspective may have important implications for future therapeutic approaches. Finally, we will discuss previous and more recent research into ILD-PH therapeutics, other management considerations, and the value of phenotyping patients with ILD-PH.

## 2. Pulmonary Hypertension

In health, pulmonary circulation is a low-pressure, low-resistance system able to accommodate the entire cardiac output with each cardiac cycle. This low-pressure state is maintained, despite increases in cardiac output and pulmonary blood flow, by a further reduction of pulmonary vascular resistance (PVR) that is achieved through the recruitment and distension of under-perfused pulmonary arteries and arterioles [13].

Pulmonary hypertension is defined by an elevated mean pulmonary arterial blood pressure (mPAP) exceeding 20 mmHg [14,15]. Right heart catheterisation (RHC) remains the gold standard for measuring pulmonary arterial pressure [15,16], while non-invasive assessment using transthoracic echocardiography (TTE) has certain limitations in detecting PH in clinical practice [17]. Various strategies using combined non-invasive assessments to predict ILD-PH have been proposed [16,18,19,20,21,22,23]; however, there is currently no widely accepted or standardised approach. Expert consensus recommends using a combination of blood tests and physiological and radiographic assessment to help identify risk before proceeding to confirmatory RHC [24].

Haemodynamic measurements not only confirm the diagnosis of PH but also offer valuable physiological and aetiological insight. These measurements are crucial for differentiating between pre-capillary, post-capillary and mixed forms of disease by using PVR and pulmonary artery wedge pressure (PAWP). Recent European guidelines classify severe PH associated with lung disease or hypoxia by a PVR > 5 mmHg [15], as this threshold is strongly associated with reduced survival [25]. These measurements enable clinicians to accurately stratify patients based on the underlying disease mechanism and suspected aetiology, as outlined in Table 1, which has important implications for management.

## 3. Pulmonary Hypertension in ILD

While PH is a well-recognised and relatively common complication of ILD, comorbidities can sometimes be the primary driver of PH, especially when parenchymal disease is limited. It is, therefore, essential to conduct a thorough assessment to identify relevant comorbidities and ensure appropriate treatment. For example, a study found that 27% of patients with idiopathic pulmonary fibrosis (IPF) and PH had post-capillary disease as diagnosed on RHC, suggesting sub-clinical left heart disease was the cause of PH rather than ILD [26]. Additionally, it is important to note that PH associated with sarcoidosis and Langerhans cell histiocytosis, both themselves causes of ILD, is classified under WHO Group 5 due to unclear or multifactorial mechanisms of PH and would therefore not necessarily be associated with pre-capillary haemodynamic measurements. This article will focus on ILD-PH, which falls under Group 3 and is defined by pre-capillary haemodynamics, comparable to Group 1 (PAH) and Group 4 (Table 1).

**Figure 1:**
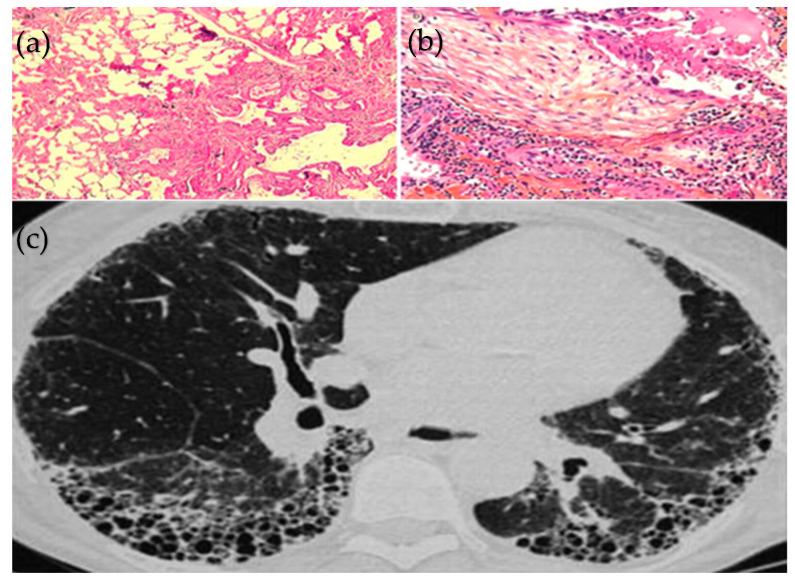
**UIP pattern on Histopathology:** Alternating regions of (**a**) relatively normal parenchyma alongside (**b**) fibroblastic foci. (**c**) **UIP on High-resolution CT:** Classic CT appearance of IPF with predominantly posterior basal, subpleural distribution with honeycombing. Reproduced with permission of the © ERS 2024: European Respiratory Review 20 (120) 108–113; DOI: 10.1183/09059180.00001611 Published 1 June 2011 [30].

Much of the available epidemiological data for ILD-PH comes from studies in the IPF population, which mostly predate recent changes to the mPAP threshold or requirement for elevated PVR [15]; the impact of these changes on the data is unknown. IPF is considered the prototypical fibrotic ILD, characterised by a usual interstitial pneumonia (UIP) pattern both histopathologically and on computerised tomography (CT) (Figure 1). Despite imperfect epidemiological data and significant geographic variability, IPF is often cited as the most prevalent ILD [27]. A systematic review has suggested that approximately 30 to 50% of patients with IPF develop clinically significant PH and that it is more commonly associated with advanced stages of disease [28], although it may occur at any stage [28,29].

The epidemiology of PH in non-IPF ILD is less well described but likely remains significant. Accurate data collection faces challenges due to small patient populations and the need for invasive RHC, which requires clinical justification. A single-centre retrospective study found that 9.5% of patients with hypersensitivity pneumonitis (HP) developed PH, although the means of diagnosis were not specified [31]. Another study conducted in patients with symptomatic fibrotic HP reported a 44% prevalence of PH confirmed by RHC [32]. A retrospective study of biopsy-proven idiopathic non-specific interstitial pneumonia (NSIP) diagnosed PH in 31.4% of patients undergoing RHC [33]. Otherwise, TTE assessment without RHC has suggested a PH incidence of 28% in idiopathic interstitial pneumonia (IIP) and 21% in connective tissue disease-associated ILD (CTD-ILD) [34], accepting the limitations of TTE assessment already discussed.

PH in IPF has been associated with a 16.7-month reduction in median survival compared with IPF alone [28]. Apart from mortality, IPF patients with just mild to moderately restrictive lung function and Group 3 PH exhibit lower resting oxygen saturations, reduced gas transfer, diminished exercise capacity, and greater desaturation with exertion than those without PH [26]. Importantly, individuals with both IPF and PH are at higher risk for acute exacerbations of IPF [35,36], which are severe and often fatal events with a mortality rate of approximately 50% [37]. Similarly, patients with fibrotic ILD complicated by PH experience increased morbidity and mortality [38,39]. Survival is significantly poorer in patients with IIP and PH compared to those with idiopathic pulmonary arterial hypertension (IPAH) alone [40], suggesting the combination of ILD and PH may be particularly harmful.

## 4. Histopathology and Pathophysiology of ILD-PH

Fibrotic ILD is thought to result from an aberrant wound-healing response arising from repeat injury to the alveolar epithelial cells (AECs). This triggers the recruitment of fibroblasts and myofibroblasts, key effector cells in pulmonary fibrosis [41]. Activated myofibroblasts produce large quantities of extracellular matrix (ECM) proteins and inflammatory and fibrogenic mediators. Transforming growth factor β1 (TGF-β1) expression and ECM accumulation further promote myofibroblast survival, leading to continued fibrogenesis [42]. Genetic factors play a significant role in both familial and sporadic cases of fibrotic ILD, with genetic variants related to DNA repair (such as *TERC* and *TERT*), host defence (like *MUC5B*) and cell–cell interactions (such as *DSP*) implicated [43,44].

Excessive ECM deposition compromises the alveolar–capillary membrane, essential for gas exchange, leading to a significant reduction in the pulmonary vasculature available for blood flow, particularly at the level of the small vessels and capillaries. Compressed or distorted pulmonary vessels from architectural disruption further elevate pulmonary vascular resistance [13]. Blood vessels are redistributed from fibrotic to non-fibrotic regions, with newly formed vessels frequently lacking elastin and exhibiting atypical morphology, leading to a further reduction in vascular compliance [45].

**Figure 2:**
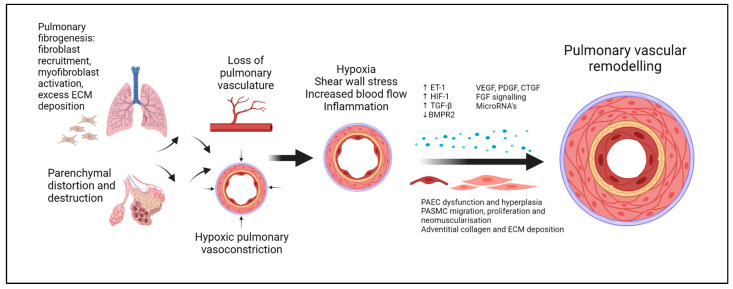
The pathophysiology of ILD-PH.

These histopathological changes disrupt normal, laminar blood flow and, when combined with shear wall stress and hypoxia, can trigger an inflammatory cascade that contributes to and perpetuates pulmonary vascular remodelling [46], a phenomenon most evident in fibrotic regions of the lung parenchyma [47]. Hypoxic pulmonary vasoconstriction (HPVS), an important homeostatic mechanism in healthy lungs, also plays an important role. The combination of HPVS and vascular remodelling can lead to vascular obliteration, further reducing the cross-sectional area available for pulmonary blood flow to participate in gas exchange (Figure 2). These structural changes have been demonstrated in vivo using CT with pulmonary vascular reconstruction in patients with IIP-PH [48].

While human tissue studies in ILD-PH are scarce, Dotan et al. [49] studied explanted lungs from ILD patients undergoing transplantation. They identified pulmonary vasculopathy in all 38 patients, severe in 42%, with 21% exhibiting plexiform lesions that are typically associated with PAH. Interestingly, they found that haemodynamic measurements taken at RHC did not correlate with the presence or severity of pulmonary arterial vasculopathy or the presence of plexiform lesions, highlighting just how prevalent pulmonary vasculopathy is in advanced ILD, despite apparently reassuring RHC measurements [49].

The physiological and clinical impact of the vascular changes described above is substantial. Most importantly, the increase in pulmonary arterial pressure causes strain and stress on the right ventricle, eventually leading to structural changes and impaired right ventricular function. Right ventricular impairment heralds a very poor prognosis, and in PAH is considered the primary determinant of functional capacity and survival [50].

## 5. Cell Signalling Pathways and Molecular Mechanisms in ILD-PH

Although the processes underlying ILD-PH are not fully understood, the role of inflammation is increasingly recognised as important. In hypoxic animal models, the degree of perivascular inflammation correlates to the degree of vascular remodelling [46]. Hypoxia alone is unlikely to be sufficient to initiate or sustain these changes; rather, a “second hit”, such as increased blood flow, shear wall stress, or additional inflammation, appears necessary for sustained pathological changes to occur [46]. These changes include intimal thickening with pulmonary artery endothelial cell (PAEC) hyperplasia, medial thickening from pulmonary artery smooth muscle cell (PASMC) proliferation, and adventitial thickening involving immune cell infiltration, fibroblast proliferation, and collagen and ECM deposition [46].

The following discussion explores various closely related cell signalling pathways and molecular mechanisms involved in the development of fibrotic ILD, PH and ILD-PH, highlighting the close and interconnected relationship in disease pathogenesis.

Endothelin-1 (ET-1) production is increased by PAECs in response to hypoxia. This peptide exhibits potent vasoconstrictive, mitogenic, pro-inflammatory, and chemoattractant properties [51]. The efficacy of endothelin receptor antagonists (ERA) in treating PAH is well-established. Increased ET-1 expression has been demonstrated in the lungs of patients with IPF [52], pulmonary fibrosis with PH [53], systemic sclerosis-ILD (SSc-ILD) [54] and the serum of patients with IPF and CTD-ILD [55]. ET-1 receptor antagonism has also been shown to reduce pulmonary fibrosis in bleomycin rat models [56]. Despite this, the clinical application of ERAs in ILD-PH has been unsuccessful with no clinical benefit demonstrated to date.

There is increasing evidence for the importance of hypoxia-inducible factor-1α (HIF-1α) in the pathogenesis of PH [57]. Increased HIF-1α expression has been observed in the remodelled pulmonary vessels of patients with IPF-PH compared to patients with IPF alone [58]. Experiments using bleomycin mouse models have suggested that HIF-1α expression may influence myofibroblast differentiation and contribute to progressive pulmonary fibrosis [59]. These data underscore the need for further research into the HIF-1α pathway in the development of ILD-PH.

HIF-1α induces vascular endothelial growth factor (VEGF) expression under low oxygen conditions. In rat models, VEGF receptor blockade combined with chronic hypoxia replicates severe PH [60]. VEGF administration has been shown to reduce endothelial apoptosis and improve pulmonary pressures however it also exacerbated pulmonary fibrosis [61]. In contrast, anti-VEGF therapy was found to reduce lung injury in transgenic mice elsewhere [62]. Despite contradictory findings in the literature, VEGF is suggested to play a role in maintaining normal lung structure and function [63], potentially aiding in alveolar wall regeneration and contributing to the vascular heterogeneity observed in IPF lungs [64]. While the exact role of VEGF in fibrotic ILD remains unclear, it is likely to play an important, if not yet well-defined, role in ILD-PH pathogenesis.

TGF-β is an important regulator of inflammation and vascular remodelling in the lung and plays a central role in PAH pathogenesis [65]. It is also the dominant pro-fibrotic cytokine responsible for myofibroblast differentiation [66] and persistent activation, contributing to the tissue damage seen in fibrotic ILD [67]. Bone morphogenetic protein receptor type II (BMPR2), a receptor belonging to the TGF-β family, is essential for regulating the growth and differentiation of various cell types, including endothelial cells. Genetic mutations of BMPR2 are the most well-documented cause of heritable PAH [68]. Notably, BMPR2 is significantly decreased in the lung tissue and macrophages of patients with IPF, particularly in those with IPF-PH [69]. Impaired BMPR2 signalling, stemming from the endothelial dysfunction and vascular remodelling observed in ILD-PH, may further exacerbate the fibrotic process [70], highlighting the close and dynamic relationship between ILD and PH pathogenesis.

Pro-fibrotic growth factors such as platelet-derived growth factor (PDGF), connective tissue growth factor (CTGF), and insulin-like growth factor (IGF-1) contribute to both parenchymal fibrosis and vascular remodelling [71]. Similarly, fibroblast growth factor (FGF) signalling affects PAECs, promotes PASMC proliferation [72] and has been observed to increase alongside HIF-1α, suggesting a likely role in hypoxic PH [73].

Epithelial and endothelial-to-mesenchymal transition (EMT and endoMT) are related processes where epithelial cells and endothelial cells differentiate into mesenchymal cells, often in response to tissue damage. These processes are important in the pathogenesis of fibrotic ILD and PAH respectively [47]. The identification of 16.2% of fibroblast-like cells from bleomycin-treated mouse lungs as endothelial in origin suggests a role for the pulmonary vascular endothelium in pulmonary fibrogenesis [74], further supporting the hypothesis of an interrelated pathogenesis.

Preclinical studies suggest that prostacyclin may exert anti-fibrotic activity in both the pulmonary vasculature and lung tissue [73]. The vasodilatory and antiproliferative effects of prostacyclin in PAH are well-established. Recent clinical data for a prostacyclin analogue in ILD-PH is promising [8,75] with a potential benefit also for fibrotic ILD [9,75] under continued investigation. This will be discussed in greater detail below.

Finally, studies examining gene expression offer valuable insights into the complex molecular mechanisms underlying ILD-PH. Distinct gene expression profiles have been identified in ILD patients with varying degrees of PH ranging from none to moderate or severe. Those with severe PH exhibit a pro-proliferative vascular remodelling phenotype, while ILD patients without PH show a predominantly inflammatory profile. Interestingly, patients with moderate PH present a mix of both inflammatory and proliferative characteristics [76]. Research into the role of non-coding gene-regulating microRNAs in ILD-PH pathogenesis, such as previous research showing elevated levels of microRNAs targeting BMPR2 expression in patients with IPF-PH compared to those with IPF alone, is also likely to be revealing [69].

This discussion illustrates the convergence of several key pathways and processes involved in both fibrotic ILD and PH pathogenesis that contribute to the vascular remodelling observed in ILD-PH (Figure 2). With the alveolar epithelium and pulmonary artery endothelium in such close physical proximity, there is increasing interest in the reciprocal influence of the pulmonary vasculature on the fibrotic parenchyma in ILD-PH disease pathogenesis [12,77,78,79,80].

## 6. Pharmacological Management of ILD-PH

Over the past two decades, several efforts have been made to apply PAH-specific therapy to ILD-PH. Table 2 provides a summary of the key randomised controlled trials conducted in the field.

To date, studies of endothelin receptor antagonists (ERAs) in ILD have been unsuccessful in achieving their primary outcomes. In the case of the ARTEMIS-IPF study, which investigated ambrisentan in IPF, the trial was terminated early due to poor efficacy and indication of harm [87]. The population in ARTEMIS-IPF likely had relatively mild physiological impairment as individuals with 5% or more honeycombing on CT were excluded, and just 10% of patients had PH on RHC prior to randomisation (mPAP > 25 mmHg and PAWP ≤ 15 mmHg). Similarly, the RISE-IIP [89] study, which investigated the use of riociguat in IIP with PH, was also terminated early due to an increase in progression events, including mortality. Ambrisentan and riociguat are therefore contraindicated in ILD-PH, and the use of ERAs other than ambrisentan is not recommended.

Early studies of the phosphodiesterase-5 inhibitor (PDE5i) sildenafil in ILD showed encouraging results. Compared to epoprostenol, sildenafil was associated with a reduction in PVR, increased arterial oxygenation, and maintained V/Q matching [93]. A subsequent study involving patients with severe PH and parenchymal lung disease (primarily COPD with only 3 IPF patients) indicated a trend towards reduced PVR and increased 6MWD [94]. Additionally, a small open-label study of sildenafil in IPF patients showed a significant mean improvement in 6MWD of 49 m after 3 months; however, the study sample size was small, and there was a high dropout rate for repeat 6MWT [95].

In a randomised controlled trial, sildenafil treatment in patients with advanced IPF (DLCO < 35% predicted) did not yield a statistically significant improvement in 6MWD at 12 weeks. Although there was a non-significant trend towards improved breathlessness, QoL, arterial oxygenation and DLCO [81], no RHC data were available for subgroup analysis. A further STEP-IPF study suggested that sildenafil might mitigate the decline in 6MWD and improve health-related QoL scores in patients with RVSD on TTE [96]. A subsequent network meta-analysis of various IPF treatments also suggested that sildenafil might improve mortality [97]. Despite these findings, studies investigating sildenafil in combination with the anti-fibrotic drugs nintedanib [82] and pirfenidone [83] did not achieve statistical significance for their primary endpoints. More recently, a large retrospective study has reported a significant survival benefit with sildenafil in ILD-PH patients, particularly those with normal right ventricular function at treatment onset [98].

Given these mixed results there remains clinical uncertainty regarding the role of sildenafil in ILD-PH. Consequently, European clinical guidelines offer a conditional recommendation based on very low-quality evidence that PDE5i treatment may be considered for severe PH associated with ILD in specialist centres [15].

The recent investigation into nitric oxide for treating ILD-PH showed promising results, with improvement in moderate to vigorous physical activity (MVPA) and stabilisation of overall activity compared to placebo [90]. Similar results were seen in patients with fibrotic ILD who require supplemental oxygen, including improved MVPA, University of California San Diego shortness of breath questionnaire (SOBQ) and St George’s Respiratory Questionnaire (SGRQ) [91]. Despite this, the phase III REBUILD study was terminated early due to its failure to meet primary endpoints, although no significant safety concerns were reported.

The large INCREASE study investigated inhaled treprostinil, a synthetic prostacyclin analogue, in patients with various fibrotic ILDs and achieved its primary outcome of increased mean 6MWD at 16 weeks compared to placebo. Significant reductions in NT-proBNP levels and decreased clinical worsening were also observed [8]. Unexpectedly, a placebo-corrected improvement in FVC was also reported, most pronounced in patients with IIP [9]. Data from the open-label extension suggested a sustained benefit with continued treatment, including a 51 mL mean increase in FVC at 64 weeks and a 31% reduction in relative risk of ILD exacerbation [75]. Ongoing phase III studies are further exploring the anti-fibrotic activity of treprostinil in IPF [10]. Treprostinil is therefore an encouraging development for both ILD-PH and potentially fibrotic ILD however further data are needed to fully evaluate its effectiveness, particularly in the real-world setting.

## 7. ILD-PH: Other Management Considerations

With the limited availability of effective therapies, managing ILD-PH should involve a comprehensive approach tailored to each individual patient’s medical comorbidities. Since PH is more common in advanced stages of ILD [28,29], although it does less frequently complicate mild disease, treatment of the underlying ILD should be prioritised to prevent progressive or severe parenchymal changes. This may be achieved through immunosuppression, anti-fibrotic therapy, or a combination of both, as appropriate.

Immunosuppressive therapy may limit the progression of ILD and, by extension, potentially reduce the risk of PH however there are currently insufficient data to directly support this [99]. There are limited data to suggest select patients with CTD-associated PAH may sustain improvements in pulmonary haemodynamics, 6MWD and symptoms with immunosuppression [100,101,102]; however, further research is needed to specifically assess the impact of immunosuppression in ILD-PH, including in patients without CTD.

The two available anti-fibrotic therapies, nintedanib and pirfenidone, have not shown improvements in breathlessness scores or prevented disease progression when used in combination with sildenafil [82,83]. Interest in whether the anti-fibrotic effect could protect against the development of PH remains. Nintedanib reduced vascular remodelling and improved pulmonary haemodynamics in rat models of PAH [103]. While echocardiographic data showed no significant difference between patients on and off nintedanib after 48 weeks [104], a case series involving ILD transplant candidates found that those taking anti-fibrotic therapy had a significantly lower mPAP and PVR. The study did not, however, provide data regarding the severity of underlying ILD [49].

Co-existing airway disease should be optimised to help prevent increased alveolar volumes and pressure from dynamic hyperinflation, which directly exerts pressure on pulmonary capillaries and further increases pulmonary vascular resistance [13]. Addressing and managing co-existing hypoventilation syndromes is important due to the physiological effects on the pulmonary vasculature [105]. Smoking has been shown to contribute to vascular remodelling and therefore cessation is of primary importance [106,107]. Finally, careful attention to diuretic therapy and fluid restriction are important strategies that may help improve short-term symptoms.

Current recommendations for oxygen therapy in PH are based on studies in patients with COPD where improvement in PVR and near complete reversal of recent pulmonary arterial pressure decline was shown [108]. A more recent study reported significant benefits of supplemental oxygen on exercise capacity in patients with PAH or chronic thromboembolic pulmonary hypertension (CTEPH) [109]. There is also evidence to support the use of nocturnal oxygen in pre-capillary PH, showing improvements in 6MWD, sleep-disordered breathing, and pulmonary haemodynamics after just one week [110].

A recent systematic review of oxygen therapy for ILD found insufficient evidence to recommend for or against long-term oxygen therapy due to the poor quality of existing studies [111]. Clinical practice is mainly informed by earlier research on supplementary oxygen in COPD patients [112,113,114]. Some studies support the use of ambulatory oxygen to improve exercise capacity [115,116] and health-related quality of life [116], with additional studies ongoing [117]. While nocturnal hypoxemia has been linked to worsening symptoms and increased mortality [118,119], the underlying cause for this remains unclear, and no interventional studies have been conducted. There is, therefore, insufficient evidence currently to support routine nocturnal oxygen therapy in ILD patients with nocturnal hypoxaemia. Despite the lack of robust evidence for oxygen therapy in ILD, expert consensus supports the use of oxygen for the management of respiratory failure, exertional desaturation and symptom palliation in cases of severe hypoxaemia [120].

Considering the evidence that oxygen therapy can impact disease progression in PH, we recommend its use in ILD-PH patients in accordance with local guidelines and ensuring there are no contraindications.

Finally, the presence of PH in ILD should prompt consideration of transplant referral where consistent with patient preferences and if their comorbidities do not disqualify them from transplant assessment. Regardless of transplant eligibility, palliative care for symptom management and care planning is of paramount importance given the significant symptom burden, functional limitation, limited treatment options, and poor prognosis associated with ILD-PH.

## 8. Phenotyping ILD-PH

The need for improved identification of distinct ILD-PH phenotypes, specifically where delineation of phenotypes has clinical value in terms of diagnosis, prognosis or treatment, has been proposed [11]. Perhaps the most important ILD-PH phenotype is the pulmonary vascular phenotype. This is characterised by PH that is out of proportion to the degree of parenchymal involvement and is associated with a markedly reduced DLCO and RV dysfunction [121,122]. The pulmonary vascular phenotype is likely to respond better to conventional PAH therapy and resembles the recently reported endophenotype associated with KDR gene mutations characterised by a low DLCO and mild parenchymal lung disease [123].

Research has shown that a PVR > 5 WU is associated with significantly reduced survival and that a PVR > 8 WU serves as an even better discriminator [25]. One group used a PVR > 5 WU to delineate severe ILD-PH and combined this with the degree of patients’ ventilatory impairment (mild if FVC > 70% predicted in IIP) to phenotype patients with Group 3 PH. Patients with severe PH and only mild ventilatory impairment had a significantly better response to PAH-specific treatment than patients with a more severely impaired FVC. These studies highlight the clinical utility of identifying patients with a pulmonary vascular phenotype for both prognostic and therapeutic purposes.

Other proposed phenotypes have included PH associated with combined pulmonary fibrosis and emphysema (CPFE), a phenotype linked to more severe PH than IPF alone, likely due to the impact of combined pathology on the pulmonary vasculature [11]. Additional phenotypes proposed include PH associated with lymphangioleiomyomatosis (LAM), post-tuberculosis (TB), and CTD-ILD [11]. Further research is needed to refine and develop additional meaningful phenotypes which could help improve our understanding and management of ILD-PH.

## 9. Conclusions

The development of PH is a critical milestone in the clinical course of ILD and is associated with significantly increased morbidity and mortality. While our understanding of the complex pathobiological processes underlying ILD-PH is incomplete, there is evidence to support an interconnected disease pathogenesis. The recent modest benefit of treatment with treprostinil reported in ILD-PH—and potentially fibrotic ILD—is not only encouraging but also lends further support to the hypothesis of an interrelated pathophysiology.

Looking forward, further investigation into the pathobiological mechanisms underlying ILD-PH with specific attention to the role of the pulmonary vasculature in pulmonary fibrogenesis is important. Improved patient phenotyping, further clinical evaluation of treprostinil in the real-world setting, and exploration of combination therapies are essential next steps. Increased recognition of the intertwined and converging nature of these two disease processes, as explored in this paper, will likely enhance both our understanding and management of this complex condition in the future.

## 10. Key Learning Points

- Clinically significant pulmonary hypertension (PH) is a relatively common complication of interstitial lung disease (ILD), more so in advanced fibrotic ILD, with significant implications for morbidity and mortality;
- While our understanding of the complex mechanisms underlying both fibrotic ILD and PH remains incomplete, the fibrotic and vascular processes are closely related and likely influence each other, further promoting the development of ILD-PH;
- Treprostinil, a nebulised prostacyclin analogue, may be beneficial for patients with ILD-PH, with ongoing investigation into its apparent anti-fibrotic effects in IPF further supporting the hypothesis of an interrelated pathophysiological process;
- Improved phenotyping of ILD-PH, such as the “pulmonary vascular” phenotype, will help advance our understanding of the condition and lead to improved clinical management.

## Figures and Tables

**Table 1:** World Health Organisation (WHO) classification of pulmonary hypertension and associated haemodynamic values.

WHO Group	Classification	Causes	mPAP (mmHg)	PAWP (mmHg)	PVR (Wood Units-WU)
Group 1 Pre-capillary	Pulmonary arterial hypertension (PAH)	1.1 Idiopathic 1.2 Heritable 1.3 Associated with drugs and toxins 1.4 Associated with connective tissue disease, HIV infection, portal hypertension, congenital heart disease, schistosomiasis 1.5 PAH with features of venous/capillary (PVOD/PCH) involvement 1.6 Persistent PH of the newborn	>20	≤15	>2
Group 2 Post-capillary	Pulmonary hypertension associated with left heart disease	1.2 Heart failure 2.2 Valvular heart disease 2.3 Congenital/acquired cardiovascular conditions leading to post-capillary PH	>20	>15	≤2
Group 3 Pre-capillary	Pulmonary hypertension associated with lung diseases and/or hypoxia	3.1 Obstructive lung disease or emphysema 3.2 Restrictive lung disease 3.3 Lung disease with mixed restrictive/obstructive pattern 3.4 Hypoventilation syndromes 3.5 Hypoxia without lung disease (e.g., high altitude) 3.6 Developmental lung disorder	>20	≤15	>2 Severe PH >5
Group 4 Pre-capillary	Pulmonary hypertension associated with pulmonary artery obstructions	4.1 Chronic thrombo-embolic PH 4.2 Other pulmonary artery obstructions	>20	≤15	>2
Group 5 Unclear or mixed	Pulmonary hypertension with unclear and/or multifactorial mechanisms	5.1 Haematological disorders 5.2 Systemic disorders 5.3 Metabolic disorders 5.4 Chronic renal failure with or without haemodialysis 5.5 Pulmonary tumour thrombotic microangiopathy 5.6 Fibrosing mediastinitis	>20	Not specified	Not specified

Modified and reproduced with permission of the © European Society of Cardiology & European Respiratory Society 2024: European Respiratory Journal 61 (1) 2200879; DOI: 10.1183/13993003.00879-2022 Published 6 January 20231.2. [15].

**Table 2:** Randomised controlled trials of PH-specific therapy in ILD-PH.

Study	Study Group	Primary Outcome	Result	Additional Information	Safety
Sildenafil STEP-IPF Zisman et al., 2010 [81]	Advanced IPF (DLCO < 35%) n = 180	Improvement in 6MWD (12 weeks)	Not met	Statistically significant improvement in paO2, DLCO, breathlessness and QoL	No concerns
Nintedanib + Sildenafil INSTAGE Kolb et al., 2018 [82]	IPF + DLCO ≤ 35% n = 274	Improvement in SGRQ score (12 weeks)	Not met		No concerns
Sildenafil + Pirfenidone SP-IPF Behr et al., 2021 [83]	Advanced IPF (DLCO < 40%p and at risk of G3PH) n = 177	Disease progression (52 weeks)	Not met	Disease progression defined as a composite of decline in 6MWD, respiratory hospitalisation and all-cause mortality	No concerns
Bosentan BUILD-2 Seibold et al., 2010 [84]	SSc-ILD n = 163	Improvement in 6MWD (52 weeks)	Not met		No concerns
Bosentan BUILD-3 King et al., 2011 [85]	IPF (<5% honeycombing on HRCT) n = 616	Improvement in 6MWD (52 weeks)	Not met		No concerns
Macitentan MUSIC Raghu et al., Million-Rousseau et al., 2013 [86]	IPF n = 178	Improvement in FVC (52 weeks)	Not met		No concerns
Ambrisentan ARTEMIS-IPF Raghu, Behr et al., 2013 [87]	IPF n = 492	Time to disease progression	Terminated early	Disease progression defined as a decline in lung function, respiratory hospitalisation or death	Increased likelihood of disease progression (27.4% vs. 17.2%) and death (7.9% vs. 3.7%)
Bosentan B-PHIT Corte et al., 2014 [88]	Fibrotic IIP + RHC confirmed PH n = 60	Reduction in PVRi ≥20% (16 weeks)	Not met		No concerns
Riociguat RISE-IIP Nathan et al., 2019 [89]	IIP + RHC confirmed PH n = 147	Improvement in 6MWD (26 weeks)	Terminated early		Higher SAE’s (37% vs. 23%) and mortality (8 vs. 3)
Nitric oxide iNO-PF Nathan et al., 2020 [90]	Fibrotic ILD n = 41	ΔMVPA and overall activity (8 weeks)	ΔMVPA met	Improvement in oxygen saturation in the study group	No concerns
Nitric oxide King et al., 2022 [91]	Fibrotic ILD n = 44	ΔMVPA, SOBQ and SGRQ scores (3 months)	n/a-exploratory	Stabilised activity levels and SOBQ and SGRQ scores compared to placebo	No concerns
Nitric oxide REBUILD [92]	ILD on LTOT, at risk of PH n = 145	Δ6MWD	Terminated early	Terminated early as not meeting primary or secondary endpoints	No concerns
Treprostinil INCREASE Waxman et al., 2021 [8]	Fibrotic ILD + RHC confirmed PH (PVR > 3) n = 326	Δ6MWD (16 weeks)	Δ6MWD met	Mean group difference: 31.12 m, ΔNT-proBNP from baseline: ↓ 15% vs. ↑ 46%, clinical worsening: 22.7% vs. 33.1%	No concerns

DLCO: diffusion capacity for carbon monoxide. 6MWD: 6-minute walk distance. MVPA: moderate to vigorous physical activity.

## Data Availability

No new data were created or analyzed in this study. Data sharing is not applicable to this article.
